# Amyloid-β and heart failure in Alzheimer’s disease: the new vistas

**DOI:** 10.3389/fmed.2025.1494101

**Published:** 2025-02-04

**Authors:** Hayder M. Al-Kuraishy, Ghassan M. Sulaiman, Hamdoon A. Mohammed, Sohaib G. Mohammed, Ali I. Al-Gareeb, Ali K. Albuhadily, Retaj A. Dawood, Amer Al Ali, Mohammed H. Abu-Alghayth

**Affiliations:** ^1^Department of Clinical Pharmacology and Medicine, College of Medicine, Mustansiriyah University, Baghdad, Iraq; ^2^Department of Applied Sciences, University of Technology, Baghdad, Iraq; ^3^Department of Medicinal Chemistry and Pharmacognosy, College of Pharmacy, Qassim University, Qassim, Saudi Arabia; ^4^Department of Pathological Analysis, College of Applied Science, Samarra University, Saladin, Iraq; ^5^Jabir ibn Hayyan Medical University, Najaf, Iraq; ^6^Department of Biology, College of Science, Al-Mustaqbal University, Hilla, Iraq; ^7^Department of Medical Laboratory Sciences, College of Applied Medical Sciences, University of Bisha, Bisha, Saudi Arabia

**Keywords:** Alzheimer’s disease, cardiovascular diseases, heart failure, pathogenesis, amyloid peptide

## Abstract

Alzheimer’s disease (AD) is the most common cause of dementia and represents 75% of all dementia types. AD neuropathology is due to the progressive deposition of extracellular amyloid-beta (A*β*) peptide and intracellular hyperphosphorylated tau protein. The accumulated Aβ forms amyloid plaques, while the hyperphosphorylated tau protein forms neurofibrillary tangles (NFTs). Both amyloid plaques and NFTs are hallmarks of AD neuropathology. The fundamental mechanism involved in the pathogenesis of AD is still elusive, although Aβ is the more conceivable theory. Aβ-induced neurodegeneration and associated neuroinflammation, oxidative stress, endoplasmic reticulum stress (ER), and mitochondrial dysfunction contribute to the development of cognitive impairment and dementia. Of note, Aβ is not only originated from the brain but also produced peripherally and, via the blood–brain barrier (BBB), can accumulate in the brain and result in the development of AD. It has been shown that cardiometabolic conditions such as obesity, type 2 diabetes (T2D), and heart failure (HF) are regarded as possible risk factors for the development of AD and other types of dementia, such as vascular dementia. HF-induced chronic cerebral hypoperfusion, oxidative stress, and inflammation can induce the development and progression of AD. Interestingly, AD is regarded as a systemic disease that causes systemic inflammation and oxidative stress, which in turn affects peripheral organs, including the heart. Aβ through deranged BBB can be transported into the systemic circulation from the brain and accumulated in the heart, leading to the development of HF. These findings suggest a close relationship between AD and HF. However, the exact mechanism of AD-induced HF is not fully elucidated. Therefore, this review aims to discuss the link between AD and the risk of HF regarding the potential role of Aβ in the pathogenesis of HF.

## 1 Introduction

Alzheimer’s disease (AD) is the most common cause of dementia and represents 75% of all dementia types (1, 2). AD neuropathology is due to the progressive accumulation of extracellular amyloid-beta (Aβ) peptide and intracellular deposition of hyperphosphorylated tau protein (3). The accumulated Aβ forms amyloid plaque, while the hyperphosphorylated tau protein forms neurofibrillary tangles (NFTs) (4, 5). Both Aβ and NFTs trigger progressive neurodegeneration directly or through the initiation of oxidative stress and inflammation (6, 7).

Aβ-induced neurodegeneration and associated neuroinflammation, oxidative stress, endoplasmic reticulum stress (ER), and mitochondrial dysfunction contribute to the development of cognitive impairment and dementia (8). Under physiological conditions, a low percentage of amyloid precursor protein (APP) is processed via *β* and *γ* secretases in the amyloidogenic pathway to produce oligomer Aβ, which is eliminated by many pathways into the systemic circulation, where it is metabolized by the liver and excreted by the kidney (9). In addition, Aβ is eliminated by cellular autophagy and degraded by enzymes such as neprilysin (NEP) and insulin-degrading enzyme (IDE). Therefore, defective autophagy and downregulation of NEP and IDE are linked with the progression of AD neuropathology (10, 11). Most of the APP processing in young healthy subjects is by *α*-secretase in the non-amyloidogenic pathway to produce the neuroprotective soluble APP alpha (sAPPα) (9). In the aging process, hypoxia, and ischemia, APP processing is shifted toward the amyloidogenic pathway (12). AD is considered an aging-related disorder due to the augmentation of oxidative stress, low-grade inflammatory reactions, and abnormal immune responses that affect APP processing and shift it toward the amyloidogenic pathway (12). Interestingly, the functional capacity of neuronal autophagy and the expression of NEP and IDE are extremely reduced by aging, leading to the impairment of Aβ elimination (10). Furthermore, an ischemic stroke increases the risk of AD and other types of dementia (13). In addition, genetic and environmental risk factors trigger the development of AD neuropathology through the induction of oxidative stress and inflammation (14).

AD occurs in two forms: early-onset familial and late-onset sporadic; genetic mutations in presenilin 1 (PS1), presenilin 2 (*PS2*), and *APP* genes cause early-onset familial AD, and a combination of lifestyle, environment, and genetic factors causes the late-onset sporadic form of the disease (15). However, accelerated disease progression is noticed in patients with familial AD. The early-onset familial AD represents 10% of all AD cases, although late-onset sporadic AD is the most common type and contributes to 90% of AD cases (16, 17). The main cause of early-onset familial AD is the overproduction of neurotoxic Aβ; nevertheless, the chief cause of late-onset sporadic AD is impairment of Aβ (18, 19).

**Figure 1:**
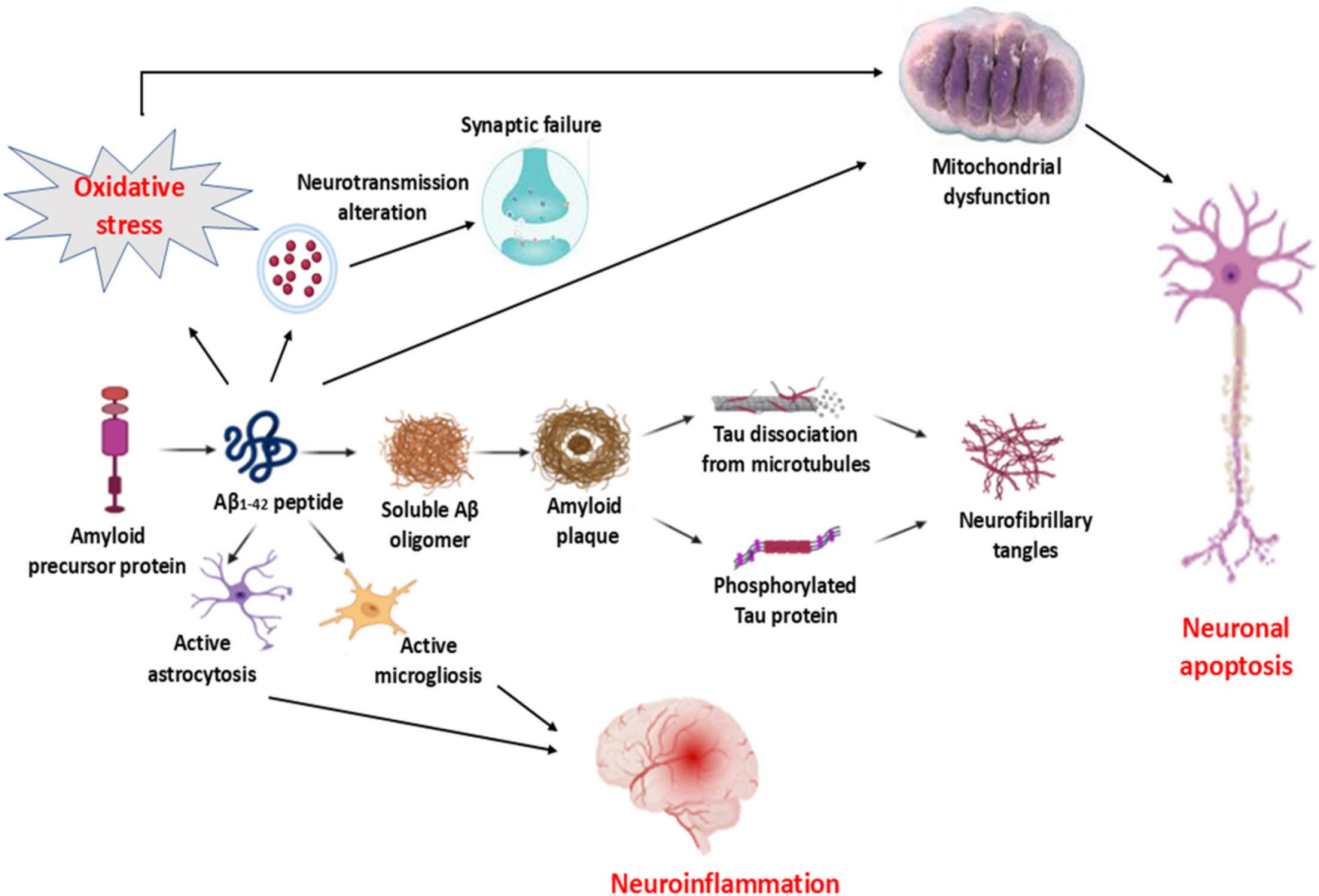
Pathophysiology of AD: Increasing processing of amyloid precursor protein (APP) to produce the neurotoxic Aβ_1-2_ is associated with the formation of amyloid plaques, which induce the dissociation of tau protein and formation of hyperphosphorylated tau protein and formation of neurofibrillary tangles (NFTs). Aβ_1-2_-induced activation of both astrocytes and microglia provokes the development of neuroinflammation. In addition, Aβ_1-2_ leads to the impairment of neurotransmitter release and the development of synaptic failure. Furthermore, Aβ_1-2_, by inducing oxidative stress, results in the development of mitochondrial dysfunction, which causes neuronal apoptosis.

Thus, the pathophysiology of AD is multifarious and related to the activation of different signaling pathways (20) (Figure 1).

**Figure 2:**
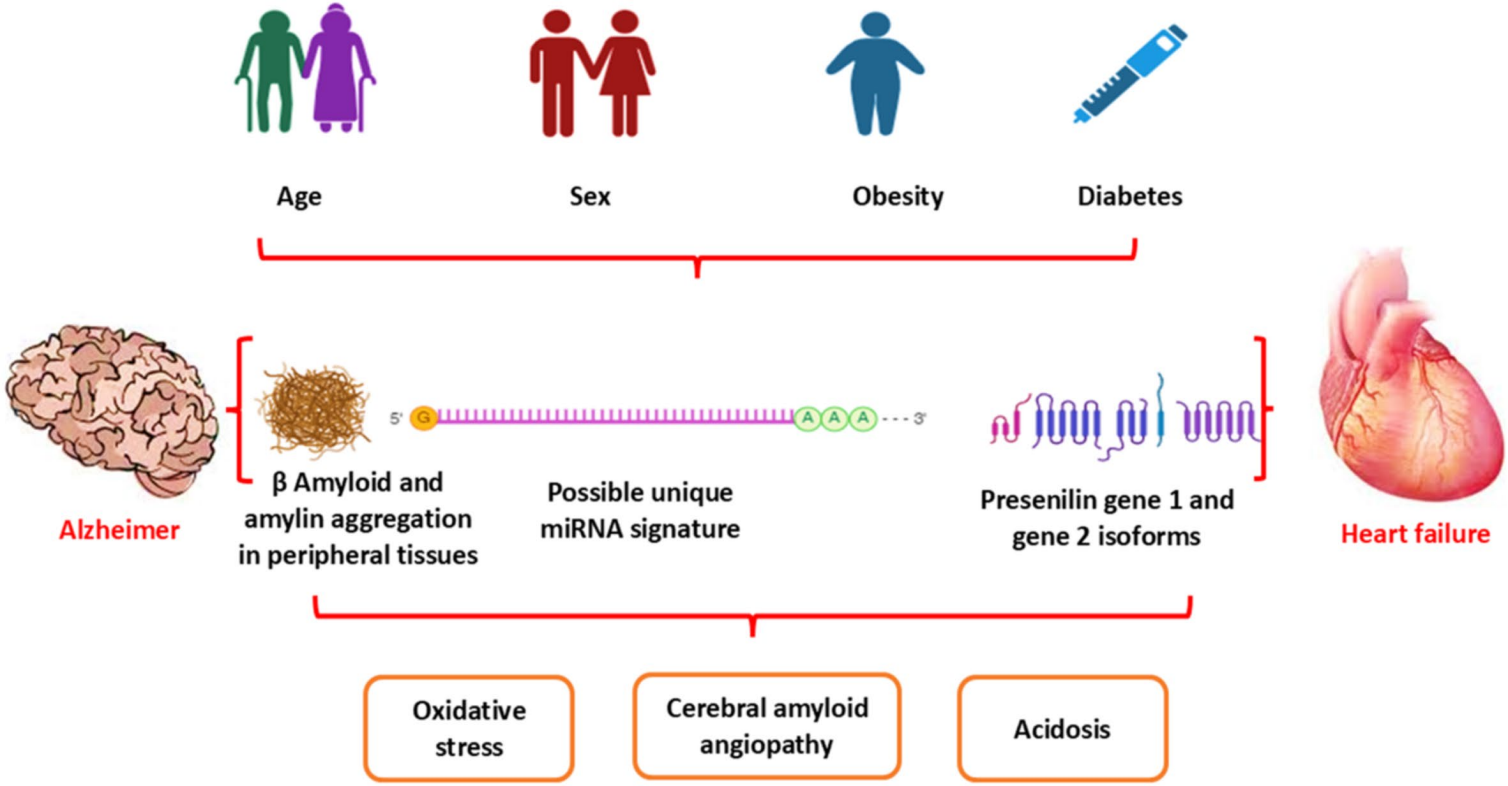
Association between AD and HF: Aging and cardiometabolic disorders, as in diabetes and obesity, provoke dysregulation of metabolic and genetic signaling, leading to the development of HF and AD. Both HF and AD are interrelated in inducing acidosis, oxidative stress, and cerebral amyloid angiopathy.

Research indicates that cardiometabolic conditions such as obesity, type 2 diabetes (T2D), and heart failure (HF) may serve as potential risk factors for the onset of Alzheimer’s disease (AD) and other dementia types such as vascular dementia (21, 22). Of note, Aβ is not only originated from the brain but also produced peripherally and, via the blood–brain barrier (BBB), can accumulate in the brain and result in the development of AD (23). HF-induced chronic cerebral hypoperfusion, oxidative stress, and inflammation can induce the development and progression of AD (12, 24). Remarkably, AD is considered a systemic disease causing systemic inflammation and oxidative stress, thereby affecting peripheral organs, including the heart (22). The development of HF due to the deposition of Aβ is called cardiac amyloidosis (13, 25). Aβ through deranged BBB can be transported into the systemic circulation from the brain and accumulate in the heart, leading to the development of HF (25). These findings suggest a close relationship between AD and HF (Figure 2). However, the exact mechanism of AD-induced HF is not fully elucidated. Therefore, this review aims to discuss the link between AD and the risk of HF regarding the potential role of Aβ in the pathogenesis of HF.

## 2 The pathophysiology of HF

HF is defined as the failure of the heart to support blood flow and the circulatory system during physical activity and rest (26). The universal definition of heart failure (HF) is a clinical syndrome characterized by symptoms and/or signs that are caused by a functional and structural abnormality of the heart, accompanied by elevated levels of natriuretic peptide and/or evidence of systemic and pulmonary congestion (27). The revised stages of HF define Stage A as at risk of HF, Stage B as pre-HF, Stage C as symptomatic HF, and Stage D as advanced HF (27). Generally, HF is classified as HF with reduced ejection fraction (HFrEF) and HF with preserved ejection fraction (HFpEF) (28).

However, the revised classification of HF proposed a new classification including HFrEF (symptomatic HF with LVEF ≤40%), HF with mildly reduced ejection fraction (HFmrEF) with LVEF ≥50%, and HF with improved ejection fraction (HFimpEF): symptomatic HF with a baseline LVEF ≤40%, a ≥ 10 point increase from baseline LVEF, and a second measurement of LVEF >40% (24). HFrEF, which typically develops subsequent to ischemic heart disease and is related to left ventricle hypertrophy, is most common in men and associated with pressure overload (29). However, HFpEF is typically developed in patients with type 2 diabetes (T2D), hypertension, obesity, and chronic kidney insufficiency (30). The pathophysiology of HFpEF differs from that of HFrEF as it is mostly attributed to coronary endothelial dysfunction and systemic inflammation (31). HFpEF is characterized by diastolic dysfunction and mild systolic dysfunction leading to increased left ventricle filling pressure (32). HFrEF, which is also called systolic HF, is mainly due to cardiomyocyte injury and defect in myocardial contractility (33).

The international prevalence of HF is in elevation due to the aging process and the obtainability of effective treatments. The prevalence of HF affects 1–2% of the general population and is expected to increase by approximately 64% from 2012 to 2030 with an equivalent increase in healthcare services by 127% (26, 34). HF is regarded as a pandemic disease affecting >64 million people globally (35). HF frequency increases with the progression of aging and reaches 10% over the age of 80 years (36). In 2022, HF will affect 64 million subjects globally with an incidence of 2% (37). The potential risk factors involved in the development and progression of HF are hypertension, ischemic heart disease, obesity, T2D, and smoking (38).

**Figure 3:**
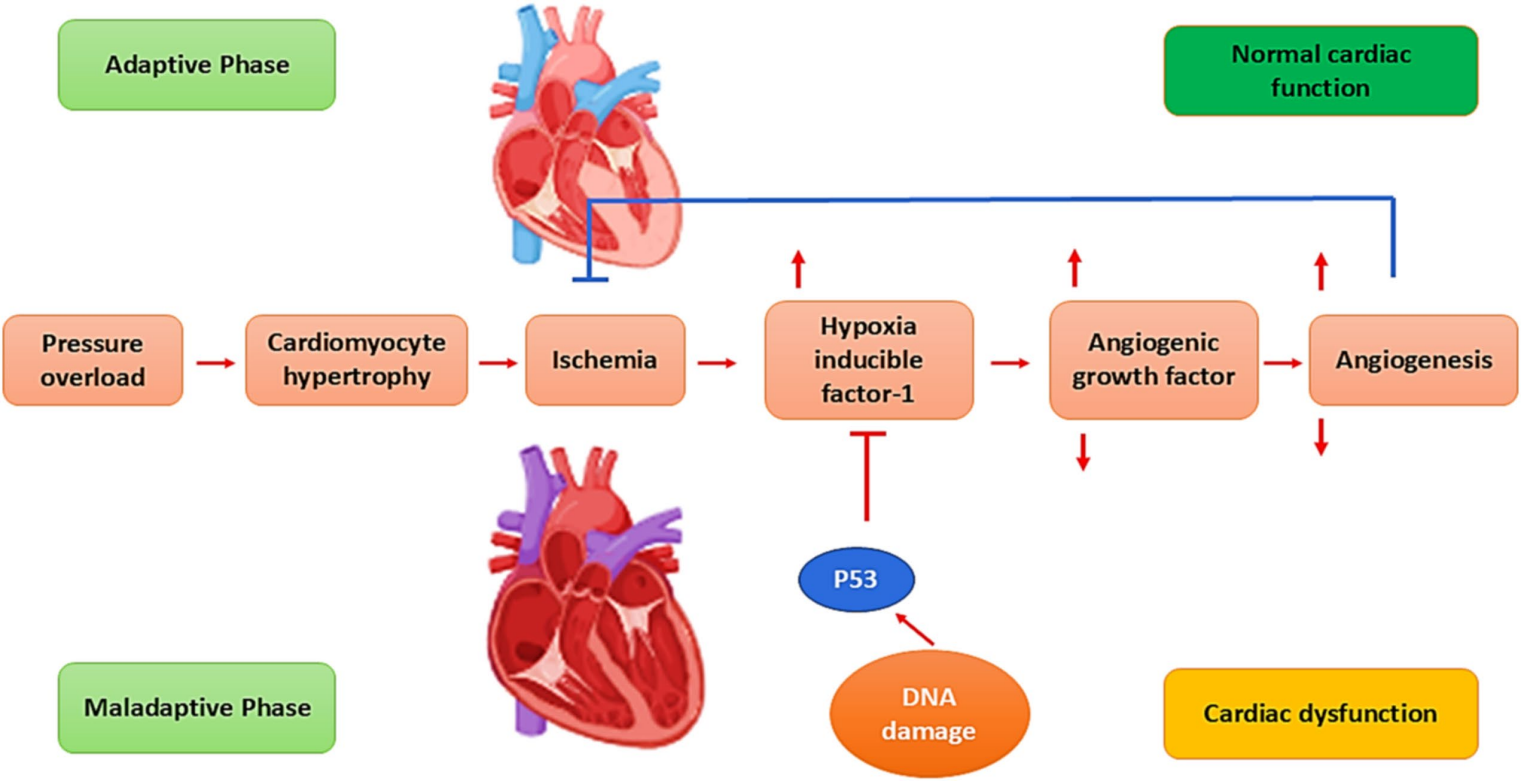
Pathophysiology of HF: Pressure overload provokes cardiomyocyte hypertrophy, which causes cardiomyocyte ischemia and promotes the release of hypoxia-inducible factor 1 (HIF-1). HIF-1 stimulates the expression of angiogenesis and angiogenic growth factors. In the adaptive phase of HF, angiogenesis induces cardiac hypertrophy to preserve cardiac function. Failure of angiogenesis prevents adaptive cardiac hypertrophy, resulting in cardiac dysfunction.

The pathophysiology of HF is related to the augmentation of preload (venous return), afterload (peripheral vascular resistance), and decreasing of cardiac contractility (26). Cardiac injury due to exaggerated preload and afterload and associated reduction of cardiac output initiates a series of neurohormonal activation such as activation of the renin–angiotensin–aldosterone system (RAAS) and sympathoadrenal system (39). These alterations affect the cardiomyocytes to compensate for the failure status. Furthermore, increasing pressure overload provokes the development of cardiomyocyte hypertrophy, which causes cardiomyocyte ischemia and promotes the release of hypoxia-inducible factor 1 (HIF-1) (40). Importantly, HIF-1 promotes the expression of angiogenic growth factors and provokes angiogenesis (41). In the adaptive phase of HF, angiogenesis induces cardiac hypertrophy to preserve cardiac function (39–41). However, lack of angiogenesis prevents adaptive cardiac hypertrophy, leading to cardiac dysfunction (42) (Figure 3).

## 3 HF and risk of AD

The association between HF and cognitive impairment was initially proposed by Emerson et al. in 1981 as cardiogenic dementia (43). Previous epidemiological studies illustrated that up to 50% of HF patients developed some degree of cognitive decline, and 10% of them experienced severe cognitive decline (44–46). However, only 26% of managed HF patients developed vascular cognitive decline (47). Prominently, the biomarkers of cardiac dysfunction and myocardial injury, such as cardiac troponin and pro-N-terminal B-type natriuretic peptide (NT-proBNP), are correlated with lower cognitive function in old patients with HF (48–50). These findings underscore the close relationship between brain and cardiac functions.

Many studies have shown that HF may increase the risk of developing AD because it lowers blood flow to the brain and raises neurohormonal activity, which leads to neuronal energy crises and neurovascular unit dysfunction (44–46, 51, 52). Long-term low blood flow to the brain from HF starts the amyloidogenic pathway, which makes neurotoxic A (2) from APP (52). An experiment showed that long-term cerebral hypoperfusion raises the levels of *β*-secretase (BACE1) and sAPP (2) while lowering the levels of *α*-secretase and sAPPα in the hippocampus of rats (52). Babusikova et al. (53) discovered that experimental global brain ischemia starts the amyloidogenic pathway of APP processing, which leads to the buildup of neurotoxic A (2). Chronic cerebral hypoperfusion leads to oxidative stress, neuroinflammation, tau protein hyperphosphorylation, Aβ buildup, synaptic dysfunction, and neurodegeneration (54). All of these factors can increase the risk of developing Alzheimer’s disease, vascular dementia, or mixed dementia. Particularly, Aβ is the key mediator for the development of cognitive impairment in aging-associated vascular pathologies (55). HF makes cognitive impairment worse because it causes chronic cerebral hypoperfusion, which damages the BBB and causes glial activation that cannot be controlled (56). Interestingly, dysregulation of the heart–brain axis seems a basic contributor to the development of cognitive impairment and dementia in HF (56).

Furthermore, exaggeration of the inflammatory and oxidative responses, injury to cerebral vasculatures, destruction of the BBB, and abnormal glial activation in HF contribute to the development and progression of AD (57–61). Research has demonstrated a link between the reduction of cardiac contractility and function, the dysregulation of the inflammatory milieu in the brain, and exaggerated sympathoexcitation (57). The HF mouse model consistently increases the expression of TLR4 and pro-inflammatory cytokines in the hippocampus and cortex (58). In addition, HF-induced oxidative stress is associated with the development of cognitive impairment in a rat model (59). Oxidative stress is a key factor in the development of Alzheimer’s disease, vascular dementia, and other neurodegenerative diseases. It does this by causing the formation of amyloid plaques and NFTs, which are characteristic of Alzheimer’s disease (60). Furthermore, HF-induced cerebral vasculature injury is associated with cerebral vascular endothelial dysfunction and the development of cognitive impairment (60). In addition, the destruction of the BBB due to chronic cerebral hypoperfusion and brain ischemia in HF triggers AD neuropathology (61). When the BBB is damaged, neurotoxic substances such as thrombin and fibrinogen can easily enter the brain from the bloodstream. This can lead to neuroinflammation and Alzheimer’s disease (62). Of interest, abnormal glial activation in HF promotes the release of pro-inflammatory cytokines and the development of neuroinflammation and AD (63, 64).

**Figure 4:**
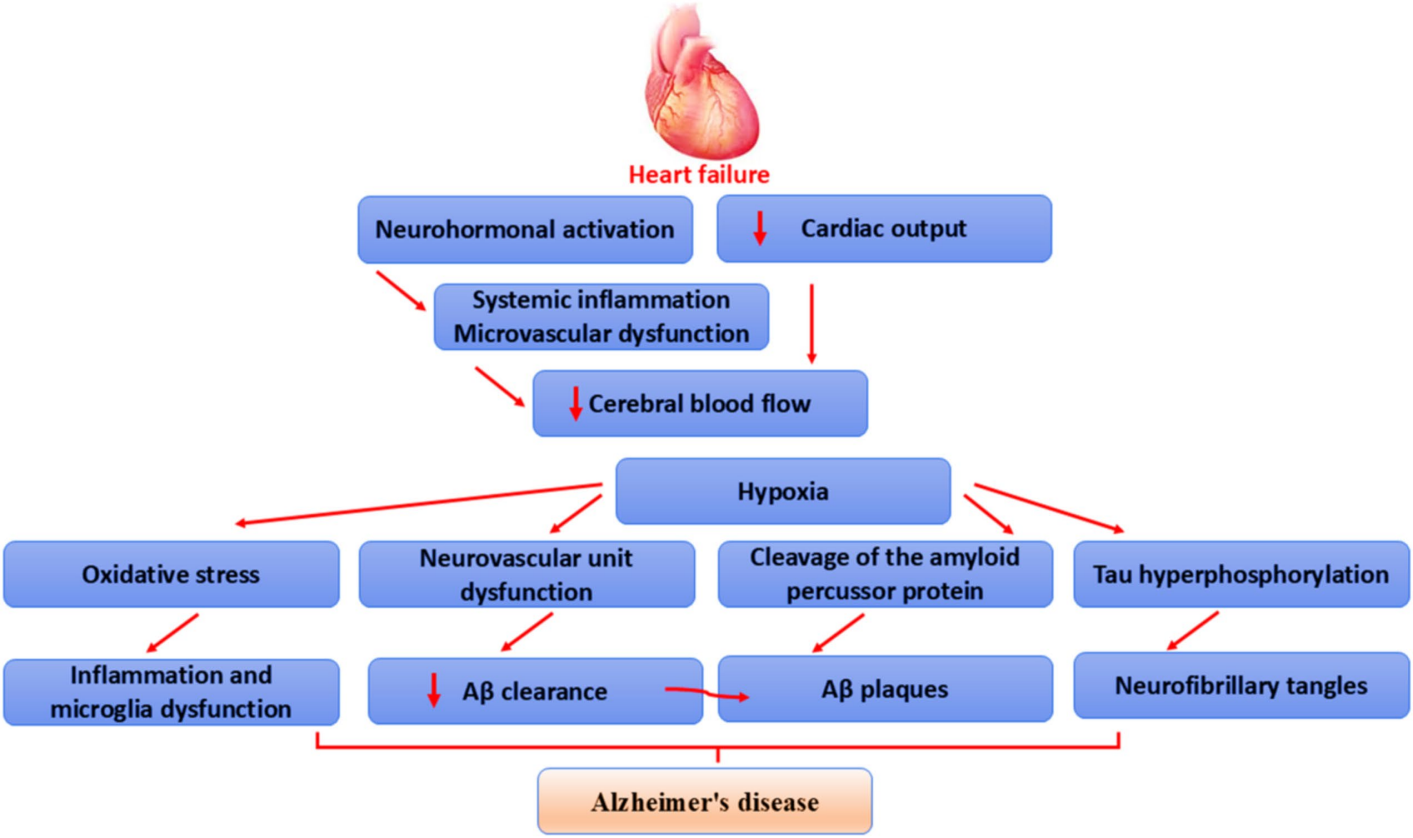
Mechanism of HF-induced AD: HF reduces cerebral blood flow through reduction of cardiac output, neurohormonal activation, systemic inflammation, and microvascular dysfunction, leading to brain ischemia. This ischemia causes tau protein hyperphosphorylation, augmentation of APP cleavage, dysfunction of neurovascular units, and oxidative stress. These changes trigger glial overactivation, inflammation, amyloid plaque formation, NFT formation, and the development of AD.

These verdicts indicated that the development of HF is influenced by abnormal inflammatory response, oxidative response, injury to cerebral vasculatures, destruction of BBB, and abnormal glial activation (Figure 4).

## 4 AD and risk of HF

**Figure 5:**
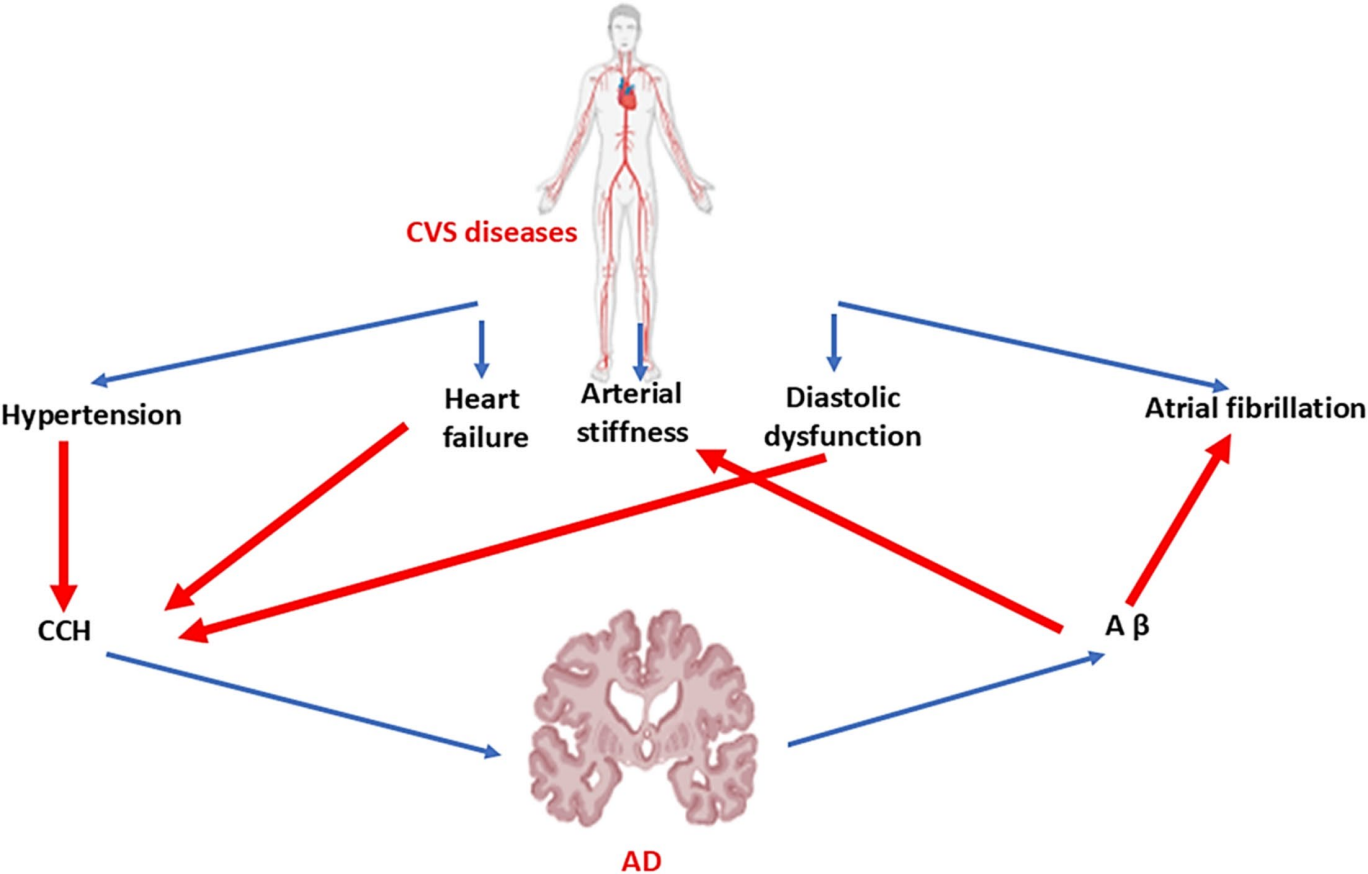
Link between cardiovascular diseases and AD: Hypertension, HF, and diastolic dysfunction lead to chronic cerebral hypoperfusion (CCH). However, AD increases peripheral Aβ, which results in the development of arterial stiffness and atrial fibrillation.

AD has been regarded as a brain-specific disease though; for decades, researchers have observed a connection between various cardiovascular abnormalities and AD, such as HF, coronary artery disease, atrial fibrillation, and vasculopathy (65). A considerable volume of work has pointed to this head-to-heart connection, focusing mainly on connotations between chronic cerebral hypoperfusion and neuronal degradation. However, new evidence of a possible systemic or metastatic profile of AD calls for further analysis of this association. Aβ aggregations are now known to be present in the hearts of individuals with idiopathic dilated cardiomyopathy as well as in the hearts of patients with AD (65–26). These findings suggest a potential systemic profile of proteinopathies and a new hypothesis for the link between peripheral and central symptoms of HF and AD. It has been shown that there is a close relationship between AD and HF. It has been shown that HF, atrial fibrillation, and hypertension through the induction of chronic cerebral hypoperfusion trigger the development of dementia and AD (65). In addition, AD increases the risk of HF and other cardiovascular disorders (65). Aβ, tau protein, and mutations of the *PSN* gene lead to arterial stiffness and diastolic dysfunction with subsequent development of HF and other cardiovascular disorders (Figure 5).

Many epidemiological studies illustrated that AD patients are associated with compromised cardiac function and early diastolic dysfunction due to the deposition of Aβ in the myocardium (66, 67). The population-based Rotterdam Study revealed that higher levels of Aβ40 were associated with worse cardiac function and a higher risk of new-onset HF in the general population (68). Interestingly, Aβ40 and Aβ42 are accumulated in the cardiomyocytes and lead to HF in AD patients compared to healthy controls (67) suggesting that AD neuropathology affects the myocardium and is implicated in the development of HF. A retrospective study illustrated that myocardial function was reduced in AD patients compared to healthy controls due to progressive accumulation of Aβ40 in HF (67). In addition, cardiac index and function are low in patients with an aging brain (66). Numerous clinical studies highlighted that aortic stiffness, impairment of cardiac diastolic dysfunction, and reduction of left ventricular ejection fraction are common in AD patients compared to healthy controls (69, 70).

An echocardiographic study revealed that AD patients exhibit diastolic dysfunction, higher atrial conduction times, and increased arterial stiffness when compared to matched healthy controls (25). A case–control study found that people with AD had lower ejection fractions and cerebral blood flow velocities, as well as higher resistance and pulsatility indices in the basilar artery, the left terminal internal carotid artery, and the right terminal internal carotid artery. The aortic and carotid arteries also had more plaques than healthy controls (26). It has been shown that the prevalence of diastolic dysfunction and QRS abnormality in AD patients was 70 and 28%, respectively (65). AD and HFpEF are common age-related disorders that can coexist (65). In addition, AD patients have more left ventricular hypertrophy and different valvular heart disease compared to healthy controls (65).

Therefore, AD may be considered a systemic disease and not limited to the brain. The relationship between AD and various physical and systemic manifestations suggests that AD is a complex disease that impacts both the central nervous system and the peripheral nervous system. Remarkably, a common feature of many systemic processes linked to AD is involvement in energy metabolism due to genetics, mitochondria, and vascular mechanisms (71). For example, mitochondrial dysfunction in AD is not isolated to neurons but occurs systemically (72). Thus, abnormal systemic changes might not only develop secondary to brain dysfunction but might also affect AD progression, suggesting that the interactions between the brain and the periphery have a crucial role in the development and progression of AD (22).

Furthermore, HF is regarded as a proteinopathy disorder due to the accumulation of Aβ in the myocardium with subsequent membrane injury and dysregulation of intracellular Ca^+2^ (73).

**Figure 6:**
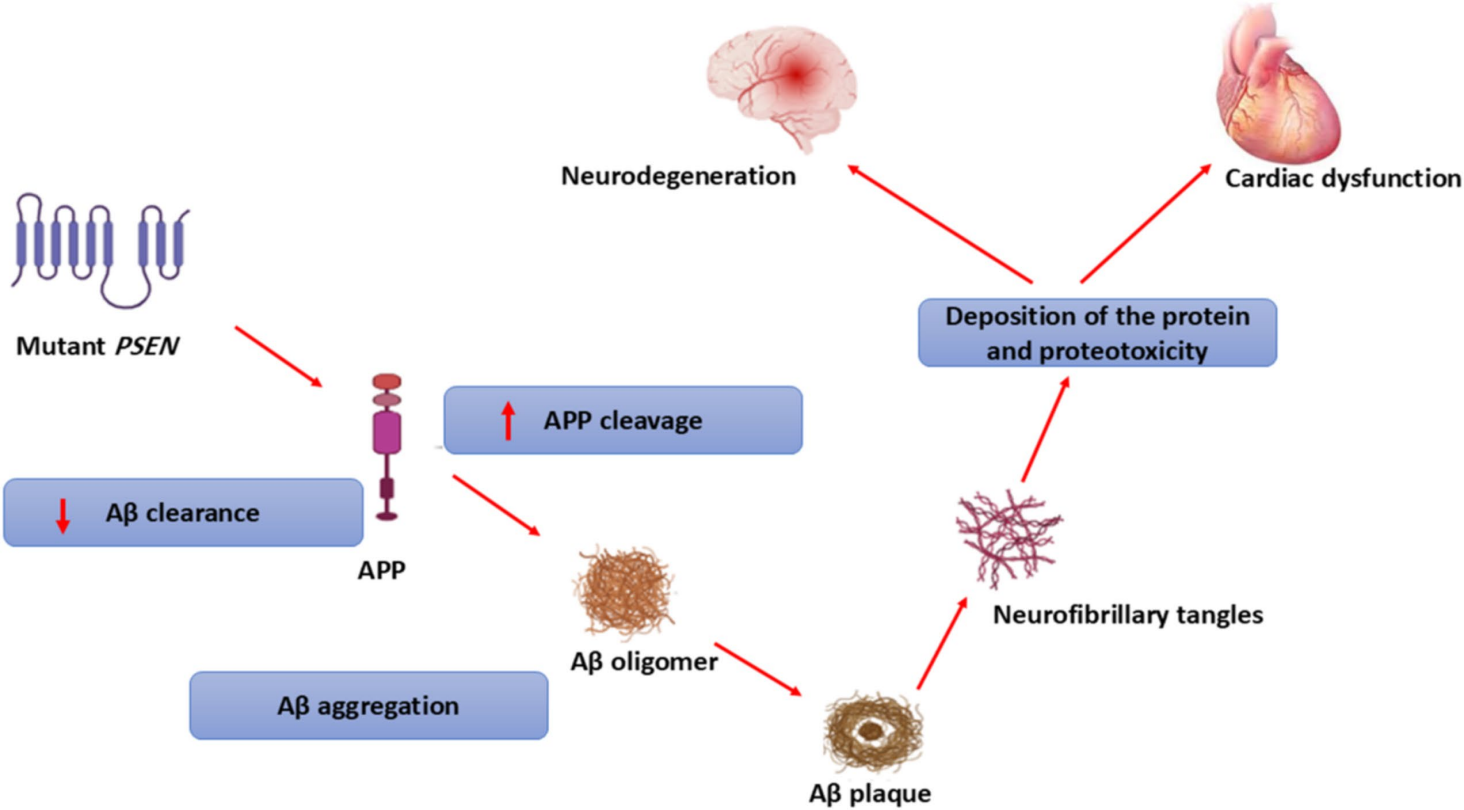
Role of *PSEN* genes in the development of neurodegenerative diseases and cardiovascular disorders.

In addition, high circulating Aβ40 levels in AD patients are correlated with the development of ischemic heart disease, a risk factor for the development and progression of HF (74). The accumulated Aβ in the myocardium of AD is much lower than in the brain (74). Of note, exaggerated of Aβ level in AD not only accumulated in the brain but also in other organ such as the heart, skin, lungs, intestine, and kidney (75). Progressive accumulation of misfolded proteins such as Aβ, wild-type transthyretin (TTR), and PSN-1 results in the development of cardiomyopathy and HF in AD patients (76). Mutations in *PSN-1* and *PSN-2* genes are associated with the development of dilated cardiomyopathy (77). However, deletion of the *PSN-1* gene is linked with the development of severe HF in mice (78) suggesting that the *PSN-1* gene is essential for the development of the heart. Mutation of the *PSN-1* gene increases the cleavage of APP and reduces Aβ clearance resulting in the formation of amyloid aggregates and NFTs in both the brain and heart (79). Therefore, *PSN* genes are regarded as a potential link in the development of neurodegenerative diseases and cardiovascular disorders (Figure 6).

Aβ aggregation in AD is associated with progressive deposition of Aβ in the heart and other peripheral tissues due to the disturbance of APP processing in the peripheral tissues (75). In addition, BBB injury in AD promotes the permeability of Aβ from the brain into the systemic circulation and deposition in peripheral tissues, including the myocardium (80). It has been shown that plasma Aβ level is correlated with brain Aβ load in AD patients (81). A systematic review and meta-analysis found that high plasma Aβ levels in elderly subjects predict the development of AD (82). Therefore, AD may increase the risk of HF by augmenting the plasma Aβ level, which is involved in the pathogenesis of cardiovascular injury and HF.

## 5 Peripheral Aβ and HF

It has been established that Aβ is also produced from peripheral tissues and implicated in the pathogenesis of AD and HF (83, 84). Citron et al. (85) found that peripheral Aβ is highly produced from skin fibroblasts of AD patients by decades before the development of symptomatic AD. Therefore, the overproduction of peripheral Aβ is involved in the pathogenesis of AD before the development of central neural abnormalities. Mounting evidence from preclinical studies illustrated that AD neuropathology can be extended systematically, causing peripheral oxidative stress and inflammation (22). Supporting this claim, left ventricular hypertrophy and aortic stenosis are developing in transgenic mice with AD model (86).

Furthermore, APP processing can occur in different cells, such as platelets, leukocytes, skeletal muscles, and cardiomyocytes, resulting in the peripheral accumulation of Aβ (87). In addition, overexpression of tau protein is associated with the development of HF and cardiac hypertrophy (22). However, the CSF level of Aβ is higher than the plasma level due to the rapid clearance of peripheral Aβ by erythrocytes (88). Unlike Aβ42, which is predominantly expressed in the brain, Aβ40 is more prominent peripherally (89). Importantly, plasma A*β* level fluctuates due to the contribution of both brain and peripheral tissues to the production of plasma Aβ level (89). The difference in the distribution of Aβ isoforms is related to the type of distributed APP in the brain and peripheral tissues. Research has shown that the brain expresses APP_695_, while leukocytes and platelets predominantly express APP_751/770_ (90). In addition, the difference in the expression of secretase enzyme (89) may explain the differences in the Aβ isoforms in the brain and periphery. The peripheral production of Aβ is augmented in HF due to the upregulation of β- and *γ*-secretase in the myocardium (91, 92). However, the expression of the cardioprotective *α*-secretase is reduced in coronary dysfunction and HF (93).

The coronary vasculature is crucial for normal heart function, especially the maturation of the coronary arterial endothelium. It has been shown that endothelial inactivation of *α*-secretase, a key regulator of Notch signaling, leads to defects in coronary arterial differentiation, as evidenced by dysregulated genes related to Notch signaling and arterial identity (93). Further analysis revealed that α-secretase knockout mice have enlarged dysfunctional hearts with abnormal myocardial compaction and increased the expression of venous and immature endothelium markers (93). These findings indicated a potential role for endothelial α-secretase in the cardioprotective homeostatic pathway.

It has been stated that the plasma Aβ42 level is augmented in patients with ischemic heart disease. A cohort study found that the plasma Aβ42 level was high in patients with non-ST elevation myocardial infarction (NSTEMI) compared to patients with STEMI (94). Therefore, the Aβ42 plasma level is value in the risk stratification of patients with NSTEMI. Importantly, plasma sAPP is increased prior to the elevation of cardiac enzymes following experimental myocardial injury (95). Therefore, endothelial APP770 is regarded as an early biomarker of acute coronary syndrome (95).

The mechanism of Aβ-induced myocardial injury is not fully elucidated, although it is related to the induction of coronary thrombosis and induction of platelet activation via the activation of the PKC signaling pathway and the production of thromboxane A2 (96, 97). In addition, Aβ40 triggers the release of MMP-9 from activated monocytes, which provokes the rupture of coronary atherosclerotic plaques (98). Consistently, high plasma Aβ is implicated in the development and progression of acute coronary syndrome by inducing thrombosis via factor XII activation, platelet activation, and monocyte activation (74, 97). Moreover, Aβ reduces the function of cardiomyocytes by inducing oxidative stress and mitochondrial dysfunction (99). Furthermore, Aβ is released from activated platelets in response to inflammation and hypoxia (100). Platelet activation is augmented in HF due to an increment in inflammation and oxidative stress (101). Plasma Aβ40 level is correlated with worsening of cardiac contractility, and a high plasma Aβ40 level predicts the development of HF (68). Plasma Aβ40 level is correlated with cardiac enzymes during myocardial injury (102). Cardiac enzymes are elevated in patients with severe HF (103); therefore, a positive correlation between plasma Aβ40 level and cardiac enzymes indicates a severe form of HF. Interestingly, plasma Aβ40 is derived from peripheral tissues, while plasma Aβ42 is derived from the brain. However, the plasma Aβ42/Aβ42 ratio is not a reliable biomarker of HF or AD (104).

Of note, > 60% of Aβ is cleared from the brain into the systemic circulation (105). In addition, peripheral clearance of Aβ is highly reduced in aging due to the impairment of renal function, and increasing efflux of Aβ across the injured BBB contributes to augmenting plasma Aβ levels (106). Therefore, both brain and peripheral tissues contribute to the elevated plasma Aβ level, which either accumulate in the peripheral organs, such as the heart or seeds back into the brain across the injured BBB (22).

Importantly, P-glycoprotein and low-density lipoprotein receptor-related protein 1 (LPR-1) mediate efflux of Aβ from the brain to the peripheral circulation (107). However, receptors for advanced glycation end product (RAGE) mediate the efflux of Aβ from the peripheral circulation to the brain (108). Moreover, the deregulation of P-glycoprotein in the myocardium reduces the clearance of Aβ40, resulting in cardiac dysfunction and the development of HF (109). Thus, impairment of the expression of P-glycoprotein and LPR-1 and overexpression of RAGE are involved in the pathogenesis of AD (107, 108, 110). It has been reported that LPR-1 plays a critical role in regulating inflammatory signaling in the myocardium and has a cardioprotective effect. Findings from preclinical studies demonstrated that LPR-1 agonists such as α1-antitrypsin reduce infarct size in acute myocardial infraction (111). Indeed, cardiac LPR-1 mediates the efflux of Aβ from the myocardium during ischemic-reperfusion injury (112). Many experimental studies highlighted that cardiac ischemic-reperfusion injury increases the expression of myocardium APP with subsequent overproduction of Aβ40, which induces the disturbance of intracellular Ca^2+^. In addition, a reduction in the expression of LPR-1 reduces the elimination of Aβ40 from the myocardium, leading to progressive myocardial injury by the initiation of inflammatory signaling (113, 114). Furthermore, RAGE is upregulated in ischemic heart disease and involved in the pathogenesis of HF. RAGE enhances the accumulation of Aβ40 in the myocardium, causing myocardial dysfunction and the development of HF (115). Upregulated RAGE is implicated in the pathogenesis of HF and other cardiovascular disorders (116).

It has been illustrated that the peripheral clearance of Aβ is by the liver, kidney, skin, and intestine, although the liver is involved in >60% of Aβ clearance (117). Similarly, the kidney eliminates plasma Aβ in a concentration-dependent manner (118). Furthermore, many blood enzymes, such as angiotensin-converting enzyme (ACE), endothelin-converting enzyme 1 (ECE-1), IDE, and NEP, are intricate in the metabolism and clearance of peripheral Aβ (119). Moreover, the soluble NEP level which is involved in the metabolism of Aβ is increased and correlated with cardiac dysfunction (120). Nevertheless, NEP is linked to the metabolism of neuronal Aβ42 rather than peripheral Aβ40 (121). Therefore, NEP inhibitors have cardioprotective effects by increasing circulating natriuretic peptides (120).

Of note, the generated Aβ from APP in the plasma is not free in the body fluids but binds different binding proteins such as albumin, antithrombin III, and lipoproteins before transport and catabolism by peripheral organs (122). In addition, peripheral cells such as erythrocytes, monocytes, macrophages, neutrophils, and lymphocytes promote the clearance of peripheral Aβ (123, 124). It has been shown that impairment of peripheral Aβ clearance is implicated in the pathogenesis of HF (125). Increasing plasma Aβ level is correlated with the severity of cardiac stress in HF patients with normal cognitive function. As well, targeting peripheral Aβ by the monoclonal antibody aducanumab may reduce the severity of myocardial dysfunction in patients with HF (125). In HF, most of the peripheral clearance mechanisms of Aβ are impaired. For example, a reduction in serum albumin, which binds 89% of plasma Aβ, is correlated with severity and mortality in patients with HF (126). Moreover, hepato-renal dysfunction is common in patients with HF (127).

These verdicts indicated that overproduction and reduced clearance of peripheral Aβ contribute to the development and progression of HF. Together, exaggerated Aβ production in Alzheimer’s disease (AD) and its transport across the damaged blood-brain barrier (BBB) augment circulating Aβ, which is implicated in the pathogenesis of heart failure (HF).

## 6 Conclusion and future perspectives

HF and cardiometabolic disorders are implicated in the development and progression of AD, vascular dementia, and other neurodegenerative diseases. In addition, AD increases the pathogenesis of HF and other cardiovascular diseases, signifying a close relationship between AD and HF.

HF-induced AD is mediated by inducing chronic cerebral hypoperfusion, oxidative stress, and inflammation that promote AD neuropathology. However, AD-induced HF is mediated by the overproduction and impairment of Aβ elimination. Impairment of cardiac diastolic dysfunction and reduction of left ventricular ejection fraction are common in AD patients. Interestingly, Aβ40 and Aβ42 are accumulated in the cardiomyocytes and lead to HF in AD, suggesting that AD neuropathology affects the myocardium and is implicated in the development of HF. In addition, myocardial function was reduced in AD patients due to progressive accumulation of A*β*40. Furthermore, high circulating Aβ40 levels in AD patients is correlated with the development of ischemic heart disease, a risk factor for the development and progression of HF. Moreover, Aβ is also produced from peripheral tissues and implicated in the pathogenesis of AD and HF. Mounting evidence illustrates that AD neuropathology can be extended systematically, causing peripheral oxidative stress and inflammation. Furthermore, APP processing can occur in different cells, resulting in the peripheral accumulation of Aβ.

The peripheral production of Aβ is augmented in HF due to the upregulation of β and *γ*-secretase in the myocardium. However, the expression of the cardioprotective *α*-secretase is reduced in coronary dysfunction and HF. Moreover, plasma Aβ42 levels are increased in patients with ischemic heart disease. Aβ reduces the function of cardiomyocytes by inducing oxidative stress and mitochondrial dysfunction. Plasma Aβ40 level is correlated with the worsening of cardiac contractility, and a high plasma Aβ40 level predicts the development of HF.

Furthermore, peripheral clearance of Aβ is highly reduced in aging due to the impairment of renal function together with increasing efflux of Aβ across the injured BBB contributes to augmenting plasma Aβ level. Therefore, both brain and peripheral tissues contribute in elevating plasma Aβ level which either accumulated in the peripheral organs such as heart or seeds back into the brain across the injured BBB.

Indeed, cardiac LPR-1 mediates the efflux of Aβ from the myocardium during ischemic-reperfusion injury. The expression of LPR-1 reduces the elimination of Aβ40 from the myocardium, leading to progressive myocardial injury by the initiation of inflammatory signaling. However, RAGE is upregulated in ischemic heart disease and is involved in the pathogenesis of HF. RAGE enhances the accumulation of Aβ40 in the myocardium, causing myocardial dysfunction and the development of HF.

Interestingly, medications used in the management of HF may affect cognitive function and AD development. Sodium-glucose co-transporter-2 (SGLT2) inhibitors, originally developed for diabetes management, are increasingly studied for their cognitive benefits and in the management of HF. The multifaceted effects and the relatively favorable side effect profile of SGLT2 inhibitors render them a promising therapeutic candidate for HF patients with cognitive impairments (128–130). Interestingly, the use of SGLT2 inhibitors in elderly patients with T2D and HFpEF improves cognitive function (131). Consistently, a prospective study revealed that empagliflozin improves cognitive and physical impairment in frail older adults with T2D and HFpEF (132). A nationwide study from Sewed found that SGLT2 inhibitors are associated with lower neurocognitive disorders in patients with HF (133). Moreover, the use of SGLT2 inhibitors has a neuroprotective effect in T2D patients with HF, reducing the incidence or progression of cognitive impairment and dementia (134). The neuroprotective effects of SGLT2 inhibitors are mediated by reducing oxidative stress and neuroinflammation, decreasing amyloid burdens, enhancing neuronal plasticity, and improving cerebral glucose utilization (131, 134). Thus, SGLT2 inhibitors, by targeting Aβ and associated neuroinflammation and oxidative stress, can mitigate both HF and AD and reduce the incidence of HF in elderly patients with AD.

Taken together, these findings point out that overproduction and reduced clearance of peripheral Aβ contributes to the development and progression of HF. In addition, the exaggeration of Aβ production in AD and transport across the damaged BBB augments circulating the Aβ level, which is implicated in the pathogenesis of HF. Therefore, AD is regarded as a potential risk factor for the development of HF, although the fundamental mechanisms need to be verified by future studies.

Accordingly, targeting peripheral Aβ by inhibiting its production or increasing its elimination may reduce the risk of HF development in AD patients. In addition, the restoration of BBB integrity by novel agents may reduce the transport of Aβ from the brain into the systemic circulation. It is interesting that RAGE inhibitors and LPR-1 activators might help stop the movement of Aβ from the brain into the bloodstream and help control HF. Therefore, we recommend further preclinical and clinical studies in this area.
